# HPOSim: An R Package for Phenotypic Similarity Measure and Enrichment Analysis Based on the Human Phenotype Ontology

**DOI:** 10.1371/journal.pone.0115692

**Published:** 2015-02-09

**Authors:** Yue Deng, Lin Gao, Bingbo Wang, Xingli Guo

**Affiliations:** 1 School of Computer Science and Technology, Xidian University, Xi'an, People’s Republic of China; 2 Institute of Software Engineering, Xidian University, Xi'an, People’s Republic of China; Huazhong University of Science and Technology, CHINA

## Abstract

**Background:**

Phenotypic features associated with genes and diseases play an important role in disease-related studies and most of the available methods focus solely on the Online Mendelian Inheritance in Man (OMIM) database without considering the controlled vocabulary. The Human Phenotype Ontology (HPO) provides a standardized and controlled vocabulary covering phenotypic abnormalities in human diseases, and becomes a comprehensive resource for computational analysis of human disease phenotypes. Most of the existing HPO-based software tools cannot be used offline and provide only few similarity measures. Therefore, there is a critical need for developing a comprehensive and offline software for phenotypic features similarity based on HPO.

**Results:**

HPOSim is an R package for analyzing phenotypic similarity for genes and diseases based on HPO data. Seven commonly used semantic similarity measures are implemented in HPOSim. Enrichment analysis of gene sets and disease sets are also implemented, including hypergeometric enrichment analysis and network ontology analysis (NOA).

**Conclusions:**

HPOSim can be used to predict disease genes and explore disease-related function of gene modules. HPOSim is open source and freely available at SourceForge (https://sourceforge.net/p/hposim/).

## Introduction

Phenotypic similarity plays an important role in different biological and biomedical applications. Previous studies prove that genes with similar phenotypes yields biological modules in terms of diseases, thus it can be used in predicting disease-causing genes [1][2]. Furthermore, it is crucial for understanding the relationships between different diseases [3].

Most current methods for measuring phenotypic similarity [4][5] are based on the Online Mendelian Inheritance in Man (OMIM) database [6] that contains textual records representing genetic disorders. However, the absence of a controlled vocabulary makes it difficult to analyze the OMIM data using a computational approach [7]. The Human phenotype ontology (HPO) [8] provides a controlled and standardized vocabulary of phenotypic abnormalities annotating all clinical entries in OMIM, which sheds light on the large-scale computational analysis of the human phenome, i.e., DECIPHER [9], ECARUCA [10] and Bridge [11].

Several tools using HPO-based semantic similarity are currently available. Phenomizer [12] is the first tool for semantic similarity search over HPO, in which users input the phenotypic abnormalities of a patient as HPO IDs, and obtain a list of diagnoses in OMIM IDs. Other tools include OwlSim [13], PhenoDigm [14], PhenomeNET/PhenomeBrowser [15] and OntoSIML [16]. The detailed comparison of HPOSim and other HPO-based tools is shown in Table 1. It can be seen from the table that most of the existing tools share one drawback: the calculations of phenotypic similarity for HPO terms, genes and diseases are not well supported. Although OntoSIML and OwlSim provide functions for calculating semantic similarity, users are required to manually input the mapping from entities (gene or disease) to HPO terms, which entails additional preprocessing effort.

**Table 1:** Comparison of HPOSim and other HPO-based tools.

**Name**	**Release Type**	**Open Source**	**Term-Term Similarity**	**Gene-Gene Similarity**	**Disease-Disease Similarity**	**Gene-Disease Similarity**	**Similarity Measures**	**Combine Methods**
HPOSim	Stand Alone (R)	√	√	√	√	×	Resnik, Lin, Jiang-Conrath, relevance, information coefficient, graph IC, Wang	Max, Mean, funSimMax, funSimAvg, BMA
Phenomizer [12]	Web	×	×	×	×	√	Resnik	symmetric, unsymmetric
OWLSim [13]#	Stand Alone (Java)	√	√	√	√	√	Jaccard, Resnik, overlap/normalized overlap, GIC	Max, Mean, BMA
PhenoDigm [14]	Web	×	×	×	×	√	Mean of Jaccard and Resnik	Max, Mean
PhenomeNET [15]	Web	×	×	√*	√	√*	simGIC	Unknown
OntoSIML [16]#	Web	×	√	√	√	√	Jaccard, simGIC, Resnik	Unknown

* PhenomeNET only supports human genes included in OMIM.

# Although OntoSIML and OwlSim provide functions for calculating semantic similarity, users are required to manually input the mapping from entities (gene or disease) to HPO terms, which entails additional preprocessing effort.

“√” means the tool provides the function and “×” means the tool does not.

In addition, there exist several tools for HPO-based enrichment analysis. OntoFUNC [17] performs functional enrichment analysis over ontologies in OWL format. It is based on FUNC [18] and users need to manually input the mapping data, which is the same as OntoSIML. STOP [19] is an online tool and can be used as a Cytoscape plug-in. It can be used in the enrichment analysis of gene sets, but does not support the analysis of disease set.

Several R packages for semantic similarity and enrichment analysis are available, including GOSim [20], GOSemSim [21], DOSim [22], DOSE [23] and topGO [24]. However, these packages mainly use gene ontology (GO) [25] and disease ontology (DO) [26]. To the best of our knowledge, there is no R package that focuses on HPO-based semantic similarity and enrichment analysis.

**Figure 1:**
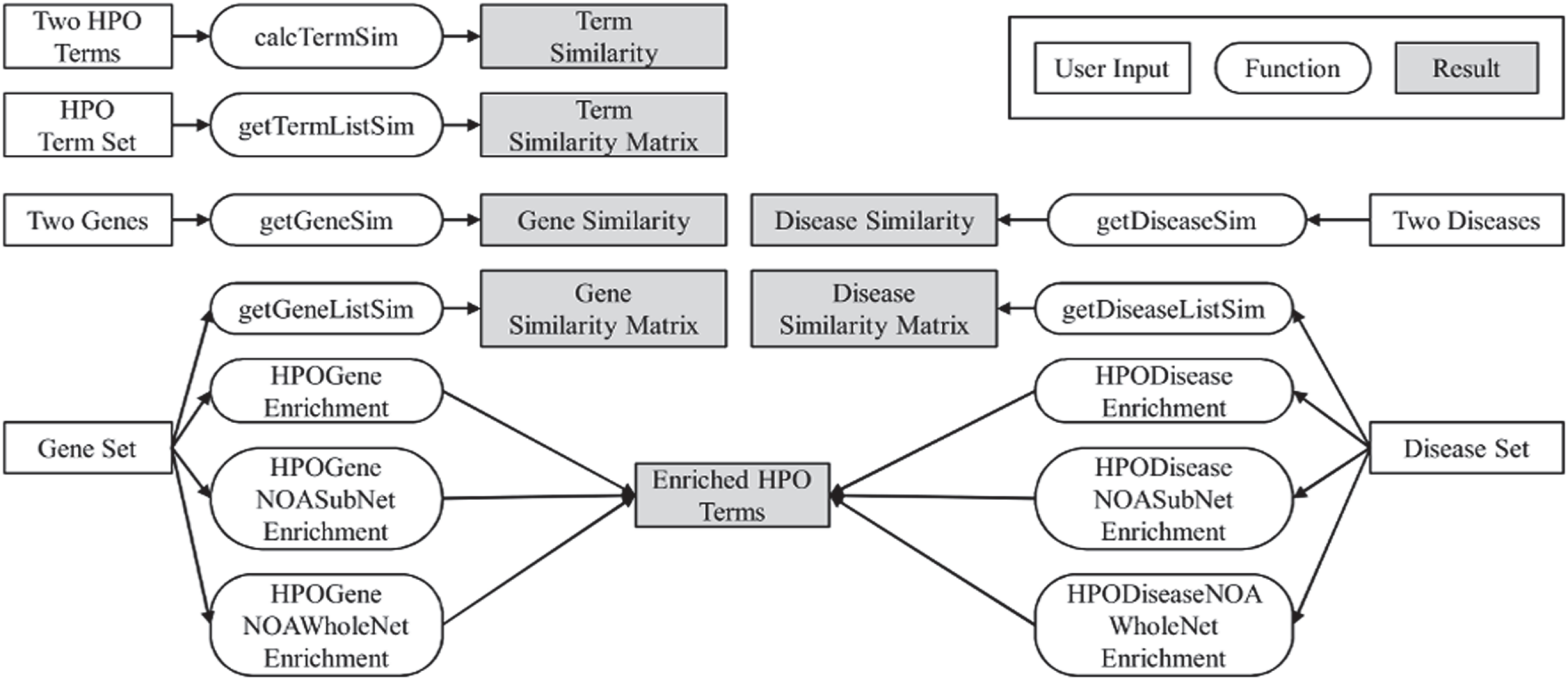
Framework of HPOSim. Users can use HPOSim to calculate semantic similarity for HPO terms, genes and diseases. HPOSim can also be used to identify enriched HPO terms for gene set and disease set.

Thus, we developed an R package HPOSim with an immediate purpose to capturing phenotypic similarities between genes and diseases. The framework of HPOSim is shown in Fig. 1. HPOSim analyzes semantic similarity for HPO terms, genes and diseases. Functional enrichment analysis of gene set and disease set are also provided, including the classic hypergeometric enrichment analysis and the novel network ontology analysis (NOA) [27].

## Implementation

### Data

**Figure 2:**
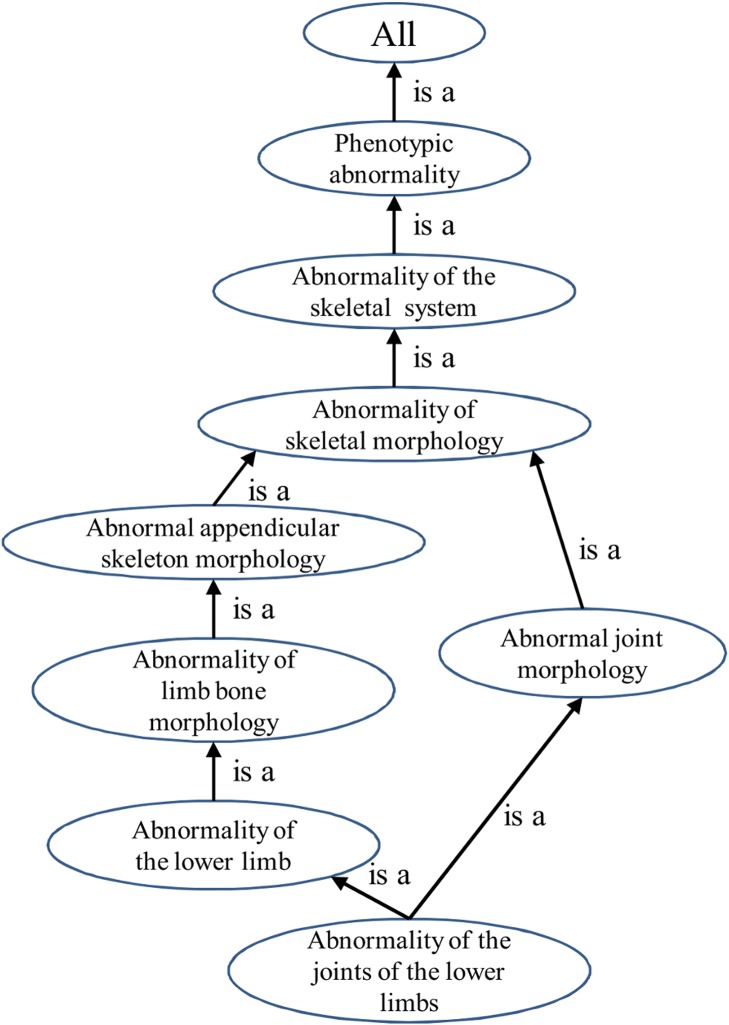
Example of the structure of HPO. HPO term *Abnormality of the joints of the lower limbs* (HP:0100491) and all its ancestor terms are shown. Each term in the HPO describes a phenotypic abnormality. Terms are related to parent terms by “is a” relationships in the form of a directed acyclic graph. If a disease or a gene is annotated to a term, it will also be annotated to all of its ancestors.

HPO contains over 10000 terms (10686 terms in the HPO build #1042 released in September 2014) in three sub-ontologies, which are phenotypic abnormality (PA), onset and clinical course (OC) and mode of inheritance (MI). Approximately 99% of the HPO terms are in the PA sub-ontology. In each sub-ontology, terms are arranged in a directed acyclic graph (DAG) and are related to their parent terms by “is a” relationships. The structure of the HPO allows a term to have multiple parent terms, which enables different aspects of phenotypic abnormalities to be explored. Diseases and genes are annotated to the most specific terms possible, which means that if a disease or a gene is annotated to a term then all of the ancestors of this term also apply (see Fig. 2 for an example).

The official ontology file provided by the HPO Consortium is in obo format, which is plain text-based. Thus, like other widely used R package for biomedical ontologies, e.g. GO.db, we constructed an R package termed HPO.db. HPO.db provided programmatic interfaces to the hierarchical structure of HPO terms. HPOSim uses HPO.db to obtain information about terms and relationships between terms. HPO.db can be used by other R packages that use HPO data.

HPOSim provides two kinds of pre-calculated data within the package: the association between HPO terms, as well as association between genes and diseases (gene-to-phenotype, phenotype-to-gene, disease-to-phenotype and phenotype-to-disease). The associations between HPO terms are obtained from the original ontology and annotation data provided by the HPO Consortium, and the information content (IC) of the HPO terms is pre-calculated based on both genes and diseases annotated to a certain term, while semantic similarity between genes and diseases are based on the IC of HPO terms.

The IC of a term *t* in HPO can be defined as follows:

IC(t)= -log(p(t)) (1)

where *p*(*t*) is the probability of observing *t* and its descendants in all genes/diseases annotated to a certain sub-ontology of HPO.

### Measuring the similarity between HPO terms

Recently, several metrics that measure the semantic similarity between ontology annotations have been proposed [28]. In HPOSim, we implement seven commonly used semantic similarity measures to measure the similarity between HPO terms: the Resnik measure [29], Lin measure [30], Jiang–Conrath measure [31], relevance measure [32], information coefficient measure [33], graph IC measure [34] and Wang measure [35]. The first six measures are based on IC, while the Wang measure uses both IC and graph structure.

The Resnik measure defines the similarity between terms as the IC of their most informative common ancestor (MICA):

$$sim_{Resnik}\left(t_{1},t_{2}\right)\text{=}IC\left(t_{MICA}\right)$$ (2)

where *t_MICA_* is the MICA of term *t_1_* and *t_2_*.

The Lin and Jiang–Conrath measures consider the IC of the two terms besides the IC of their MICA:

$$sim_{Lin}\left(t_{1},t_{2}\right)\text{=}\frac{2\times IC\left(t_{MICA}\right)}{IC\left(t_{1}\right)IC\left(t_{2}\right)}$$ (3)

$$sim_{JC}\left(t_{1},t_{2}\right)\text{= 1-}\left(IC\left(t_{1}\right)\text{+}IC\left(t_{2}\right)-2\times IC\left(t_{MICA}\right)\right)$$ (4)

The relevance measure and the information coefficient measure are based on Lin’s measure:

$$sim_{Rel}\left(t_{1},t_{2}\right)\text{=}sim_{Lin}\left(t_{1},t_{2}\right)\times \left(1-p\left(t_{MICA}\right)\right)$$ (5)

$$sim_{IC}\left(t_{1},t_{2}\right)\text{=}sim_{Lin}\left(t_{1},t_{2}\right)\times \left(1-\frac{1}{1+IC\left(t_{MICA}\right)}\right)$$ (6)

The graph IC measure takes all the common ancestors of the two terms into account:

$$sim_{GraphIC}\left(t_{1},t_{2}\right)\text{=}\frac{\sum_{t\in \left(A\left(t_{{}_{1}}\right)\cap A\left(t_{{}_{2}}\right)\right)}IC\left(t\right)}{\sum_{t\in \left(A\left(t_{{}_{1}}\right)\cup A\left(t_{{}_{2}}\right)\right)}IC\left(t\right)}$$ (7)

where *A*(*t*) is the ancestors of term t in HPO.

The Wang measure is based on the graph structure of HPO DAG. In Wang’s measure, a weight is given to each edge according to its type. *DAG_t_* = (*t*,*T_t_*,*E_t_*) represents the subgraph made up of term *t* and its ancestors, where *T_t_* is the set of the ancestor terms of *t* and *E_t_* is the set of edges in *DAG_t_*.

In *DAG_t_*, *S_t_*(*n*) measures the semantic contribution of term *n* to term *t*, which is defined as:

$$\{\begin{array}{c} S_{t}\left(t\right)=1\\ S_{t}\left(n\right)=\max\left\{w_{e}*S_{t}\left(n^{'}\right)|n^{'}\in childrenof\left(n\right)\right\}\text{if}t\neq n \end{array}$$ (8)

The similarity between HPO term *t_1_* and term*t_2_* is defined as:

$$sim_{Wang}\left(t_{1}\text{,}t_{2}\right)\text{=}\frac{\sum_{t\in T_{t_{1}}\cap T_{t_{2}}}S_{t_{1}}\left(t\right)\text{+}S_{t_{2}}\left(t\right)}{SV\left(t_{1}\right)\text{+}SV\left(t_{2}\right)}$$ (9)

where *SV*(*m*) is the sum of the semantic contributions of all the terms in *DAG_m_*.

### Combining term-term similarity into gene-gene and disease-disease similarity

In HPOSim, the similarity between two genes is calculated based on the pairwise similarity of the two HPO term sets annotating these two genes. HPOSim provides five methods to combine multiple term-term similarities into one gene-gene similarity, which are “Max” [36], “Mean” [36], “funSimMax” [32], “funSimAvg” [32], and “BMA” [35].

Given gene *g_1_* annotated by HPO term set *HPO_1_* = {*t_11_*, *t_12_*,…,*t_1m_*} and *g_2_* annotated by *HPO_2_* = {*t_21_*, *t_22_*,…,*t_2n_*}. The similarity matrix *S*=[*s_i j_*]_*m*×*n*_ contains all pairwise similarity scores of terms in *HPO_1_* and *HPO_2_*.

The “Max” method calculates the maximum semantic similarity score over all pairs of HPO terms in the two term sets, and is defined as follows.

$$Sim_{Max}\left(g_{1},g_{2}\right)=\underset{1\leq i\leq m,1\leq j\leq n}{\max}s_{ij}$$ (10)

The “Mean” method calculates the average semantic similarity score over all pairs of HPO terms in the two term sets, and is defined as follows.

$$Sim_{Mean}\left(g_{1},g_{2}\right)=\frac{1}{m\times n}\sum_{i=1}^{m}\sum_{j=1}^{n}s_{ij}$$ (11)

The “funSimMax”, “funSimAvg” and “BMA” methods are based on the maximum value in each row and column of similarity matrix *S*.

The “funSimMax” and “funSimAvg” methods [32] use the arithmetic maxima and average between similarities for two directional comparisons of the similarity matrix *S*.

$$Sim_{funSimMax}\left(g_{1},g_{2}\right)=\max\left\{\frac{1}{m}\sum_{i=1}^{m}\underset{1\leq j\leq n}{\max}s_{ij},\frac{1}{n}\sum_{j=1}^{n}\underset{1\leq i\leq m}{\max}s_{ij}\right\}$$ (12)

$$Sim_{funSimAvg}\left(g_{1},g_{2}\right)=\frac{1}{2}\times \left(\frac{1}{m}\sum_{i=1}^{m}\underset{1\leq j\leq n}{\max}s_{ij}+\frac{1}{n}\sum_{j=1}^{n}\underset{1\leq i\leq m}{\max}s_{ij}\right)$$ (13)

The “BMA” method uses the best-match average strategy, which calculates the average of all maximum similarities on each row and column of the similarity matrix *S*.

$$Sim_{BMA}\left(g_{1},g_{2}\right)=\frac{\sum_{i=1}^{m}\underset{1\leq j\leq n}{\max}s_{ij}+\sum_{j=1}^{n}\underset{1\leq i\leq m}{\max}s_{ij}}{m+n}$$ (14)

The calculation of the similarity between diseases is the same as that between genes. The similarity between two diseases is calculated based on the pairwise similarity of the two term sets annotating these two diseases.

### HPO-based Enrichment Analysis

HPOSim provides HPO-based enrichment analysis to investigate the phenotypic features of gene sets or disease sets. Two enrichment analysis methods are provided: hypergeometric test and the NOA method [27].

Given an HPO term *t* and a gene set with *T* genes, assuming that there are *R* genes/diseases annotated in the whole HPO in which *G* genes/diseases are annotated to *t*. In addition, there are *O* genes/diseases in the gene set that are annotated to *t*. The hypergeometric enrichment p-value for *t* is calculated as follows:

$$p-value\text{=}\sum_{x=O}^{\min\left(G,T\right)}\frac{\left(\begin{array}{c} G\\ x \end{array}\right)\left(\begin{array}{c} R-G\\ T-x \end{array}\right)}{\left(\begin{array}{c} R\\ T \end{array}\right)}$$ (15)

In NOA, users input a gene or disease network. For each edge in the network, the HPO terms annotating this edge are defined as the intersection of the two term sets annotating the two nodes of the edge. NOA uses HPO terms annotating the edges to perform the enrichment analysis. Two alternative strategies, “sub-net” and “whole-net”, are applied to choose the reference set. In the “sub-net” strategy, users are required to provide the reference set. While in the “whole-net” strategy, the complete graph on the nodes of the input network is used as the reference set.

## Results and Discussion

HPOSim consists of two parts: (i) the similarity measures between phenotypes (HPO terms), between human genes (Entrez IDs) and between diseases (OMIM IDs), and (ii) HPO-based enrichment analysis (NOA and the hypergeometric method) for gene set and disease set.

### Application on gene similarity and gene set enrichment analysis

We used the aging network [37] to demonstrate the application of gene semantic similarity provided by HPOSim. The aging network was constructed by identifying genes related to aging and adding edges between interacting gene pairs. After removing the genes that are not annotated in the PA sub-ontology of HPO, 102 genes and 293 interactions were remained (see S1 Dataset for detail).

First, the semantic similarity matrix of the 102 genes was constructed using the Resnik measure and “funSimMax” combining method (see S2 Dataset for detail). A hierarchical clustering was then performed using the R package stats, and six modules were detected using the R package dynamicTreeCut. HPO enrichment analysis (hypergeometric test) was then performed using HPOSim. GO enrichment analysis and pathway enrichment analysis based on KEGG (Kyoto Encyclopedia of Genes and Genomes) pathway database [38] were performed using DAVID [39]. The results are shown in Table 2.

**Table 2:** Gene modules of the aging network.

**Module**	**Size**	**Genes (Entrez ID)**	**TOP 5 Enriched GO BP Terms**	**TOP 5 Enriched HPO Terms**	**TOP 5 Enriched KEGG Pathways**
M1	36	25, 207, 472, 581, 596, 641, 672, 675, 701, 1029, 1050, 1499, 1956, 2064, 2308, 3265, 4193, 4292, 4609, 5159, 5422, 5728, 5781, 5925, 6794, 7015, 7157, 7486, 9184, 1385, 7153, 627, 1649	regulation of apoptosis, cell cycle process, regulation of programmed cell death, regulation of cell death, regulation of cell cycle	Neoplasm, Neoplasm by anatomical site, Neoplasm by histology, Sarcoma, Hematological neoplasm	Pathways in cancer, Prostate cancer, Endometrial cancer, Glioma, Bladder cancer
M2	26	545, 1387, 2010, 2033, 2068, 2073, 2074, 2260, 3479, 3480, 4000, 4036, 4792, 4803, 5979, 7020, 7314, 7341, 7415, 7507, 5830, 1950, 1161, 847, 1490, 2067	DNA metabolic process, response to UV, response to radiation, DNA repair, nucleotide-excision repair	Intrauterine growth retardation, Aplasia/Hypoplasia of the mandible, Micrognathia, Defective DNA repair after ultraviolet radiation damage, Abnormality of the mandible	Nucleotide excision repair, Prostate cancer, Pathways in cancer, Melanoma, Adherens junction
M3	17	367, 2099, 2353, 2690, 2908, 3630, 3643, 3952, 3953, 5449, 5578, 6777, 7040, 8626, 8820, 2688, 5626	response to hormone stimulus, response to endogenous stimulus, response to organic substance, positive regulation of macromolecule metabolic process, response to estrogen stimulus	Abnormality of the anterior pituitary, Abnormality of the pituitary gland, Abnormality of the endocrine system, Abnormality of the hypothalamus-pituitary axis, Anterior hypopituitarism	Jak-STAT signaling pathway, Neuroactive ligand-receptor interaction, Cytokine-cytokine receptor interaction, Aldosterone-regulated sodium reabsorption, Pathways in cancer
M4	11	355, 2071, 3561, 3575, 4683, 4791, 5295, 5580, 6774, 6929, 5336	cell activation, B cell activation, lymphocyte activation, leukocyte activation, immune system development	Abnormality of lymphocytes, Abnormal immunoglobulin level, Abnormality of B cell physiology, Abnormality of B cells, Abnormality of humoral immunity	Pathways in cancer, Jak-STAT signaling pathway, Fc epsilon RI signaling pathway, Fc gamma R-mediated phagocytosis, Neurotrophin signaling pathway
M5	9	3064, 4001, 4137, 5155, 6872, 6908, 5663, 6647, 1938	negative regulation of neuron apoptosis, regulation of neuron apoptosis, positive regulation of MAP kinase activity, behavior, regulation of membrane potential	Abnormality of extrapyramidal motor function, Personality changes, Adult onset, Dysarthria, Parkinsonism	Huntington’s disease, Basal transcription factors
M6	5	348, 351, 3717, 2876, 5328	regulation of response to external stimulus, induction of apoptosis, induction of programmed cell death, positive regulation of apoptosis, positive regulation of programmed cell death	Long-tract signs, Abnormal bleeding, Abnormalities of the peripheral arteries, Arterial stenosis, Cerebral inclusion bodies	N/A*

* N/A indicates that there are no enriched KEGG pathway (p-value<0.05) for this module.

Module M5 only have two enriched KEGG pathway (p-value<0.05).

Gene FOXO4 (Entrez ID: 4303) could not be grouped into a certain module.

It can be seen that the enriched GO and HPO annotations are largely different among these modules. For example, the enriched GO annotations of module M2 implied that aging is associated with radiation including ultraviolet (UV), which has been verified by previous study in skin aging [40]. While the enriched GO annotations of module M3 implied that aging is associated with hormone stimulus, and literature mining showed that older women require a greater parathyroid hormone stimulus than younger women [41]. The enriched HPO annotations of the module M3 implied that aging are associated with abnormality of the pituitary, which has been verified by Sano *et al*. [42]. Disease enrichment analysis based on OMIM was then performed on genes in M3 using DAVID [39] and showed that term “Pituitary hormone deficiency, combined” was representative (p-value = 8.2E-3).

The enriched pathways of different modules are closely related to cancer, however various among different modules. Jak-STAT signaling pathway was found to be representative in modules M3 and M4. In a previous study by Fulop *et al*. [43], it was found that the signalling of IL-2 receptors is altered in T cells and macrophages with aging, mainly in relation to the Jak-STAT pathway.

These results above indicate that HPO-based semantic similarity can provide a different aspect in disease-related studies other than GO.

NOA and hypergeometric gene set enrichment analysis were then performed on the aging network. The “whole-net” strategy [27] was used to choose the reference set in NOA. The top 10 enriched HPO terms in the two enrichment methods are shown in Table 3. It can be seen that both enrichment methods identify neoplasm-related HPO terms as the top hits. However, these two methods give different terms and different ranks of terms. When dealing with gene/disease sets from biological networks, users are suggested to use the NOA method. If the gene sets are not from network data, users can use either hypergeometric or NOA enrichment method.

**Table 3:** Top 10 enriched HPO terms by the NOA method and hypergeometric enrichment.

**Rank**	**NOA(whole-net)**			**Hypergeometric Enrichment**		
	**HPO ID**	**Description**	**q-value**	**HPO ID**	**Description**	**q-value**
1	HP:0011793	Neoplasm by anatomical site	<1E-14	HP:0002664	Neoplasm	<1E-14
2	HP:0002664	Neoplasm	4.8E-14	HP:0011792	Neoplasm by histology	1.2E-13
3	HP:0007379	Neoplasm of the genitourinary tract	1.6E-5	HP:0011793	Neoplasm by anatomical site	1.1E-12
4	HP:0001156	Brachydactyly syndrome	4E-5	HP:0100242	Sarcoma	3.1E-10
5	HP:0010787	Genital neoplasm	5.1E-5	HP:0004377	Hematological neoplasm	6.9E-8
6	HP:0008069	Neoplasm of the skin	5.7E-4	HP:0000008	Abnormality of female internal genitalia	7.7E-7
7	HP:0001909	Leukemia	3.6E-3	HP:0004375	Neoplasm of the nervous system	7.7E-7
8	HP:0000006	Autosomal dominant inheritance	4.2E-3	HP:0002665	Lymphoma	7.7E-7
9	HP:0000008	Abnormality of female internal genitalia	4.2E-3	HP:0000812	Abnormal internal genitalia	8.2E-7
10	HP:0000812	Abnormal internal genitalia	4.4E-3	HP:0010460	Abnormality of the female genitalia	8.6E-7

Both enrichment methods identify HPO terms related to neoplasm as the top hits. However, these two methods give different enriched terms and different ranks of terms.

### Application on disease similarity and disease set enrichment analysis

HPOSim can also be used to investigate the phenotypic relationships between diseases. First, 115 cancer related entries were obtained by searching the OMIM database [6] using “cancer” or “carcinoma” as the key word. After removing the diseases that are not annotated in the PA sub-ontology of HPO and all the genes, 55 disease entries were remained (see S3 Dataset for detail).

The semantic similarity matrix of the 55 disease entries was constructed using the Resnik measure and “funSimMax” combining method (see S4 Dataset for detail). A hierarchical clustering was then performed and four modules were detected using the same routine as used in the previous case study. HPO enrichment analysis (hypergeometric test) was also performed using HPOSim. The results are shown in Table 4.

**Table 4:** Disease modules of the cancer entries in OMIM.

**Module**	**Size**	**Diseases (OMIM ID)**	**TOP 5 Enriched HPO Terms**
M1	22	OMIM:246470, OMIM:114550, OMIM:120435, OMIM:133239, OMIM:137215, OMIM:148500, OMIM:260350, OMIM:276300, OMIM:601228, OMIM:606719, OMIM:608615, OMIM:609310, OMIM:612229, OMIM:612591, OMIM:613244, OMIM:613347, OMIM:613659, OMIM:614331, OMIM:614337, OMIM:614350, OMIM:614385, OMIM:615083	Neoplasm by anatomical site, Neoplasm, Abnormality of the large intestine, Neoplasm of the large intestine, Neoplasm of the gastrointestinal tract
M2	13	OMIM:109400, OMIM:109800, OMIM:114500, OMIM:144700, OMIM:150800, OMIM:176807, OMIM:273300, OMIM:300854, OMIM:312300, OMIM:601518, OMIM:603688, OMIM:605074, OMIM:608089	Neoplasm of the genitourinary tract, Neoplasm, Neoplasm by anatomical site, Genital neoplasm, Urinary tract neoplasm
M3	12	OMIM:603641, OMIM:114480, OMIM:158320, OMIM:167000, OMIM:211980, OMIM:260500, OMIM:275355, OMIM:603956, OMIM:604370, OMIM:612555, OMIM:614456, OMIM:614564	Breast carcinoma, Neoplasm, Neoplasm of the breast, Neoplasm by anatomical site, Abnormality of the breast
M4	6	OMIM:155240, OMIM:171400, OMIM:188470, OMIM:188550, OMIM:202300, OMIM:608266	Neoplasm of the endocrine system, Thyroid carcinoma, Neoplasm of the thyroid gland, Abnormality of thyroid morphology, Neoplasm by anatomical site

OMIM:191600 (URETER, CANCER OF) and OMIM:610644 (PALMOPLANTAR HYPERKERATOSIS WITH SQUAMOUS CELL CARCINOMA OF SKIN AND 46,XX SEX REVERSAL) could not be grouped into a certain module.

The results showed that these four disease modules had different phenotypic features. For example, module M3 included several types of women-only cancer, including breast cancer (OMIM:114480), breast-ovarian cancer (OMIM:604370, OMIM:612555), ovarian cancer (OMIM:167000) and cervical cancer(OMIM:603956). And lung cancer (OMIM:211980) in M3 was the second most commonly diagnosed types of cancer among women in 2013[44].

The result above indicated that HPO-based semantic similarity had potential ability to play an important role in disease classification and other disease-related studies.

## Conclusions

HPOSim is an open source R package that contains seven semantic similarity measures and two enrichment analysis based on HPO data. Also, it provides useful functions for disease-related research and can be integrated with other R packages. In future work, we will integrate more similarity measures and other functions, such as visualization of the HPO data.

## Supporting Information

S1 Dataset Aging network after removing the genes that are not annotated in PA sub-ontology of HPO. (CSV)

S2 Dataset Semantic similarity matrix of the 102 genes in the aging network. (CSV)

S3 Dataset Cancer entries in OMIM. (XLSX)

S4 Dataset Semantic similarity matrix of the 55 cancer entries. (CSV)
